# Poke Weed Mitogen Requires Toll-Like Receptor Ligands for Proliferative Activity in Human and Murine B Lymphocytes

**DOI:** 10.1371/journal.pone.0029806

**Published:** 2012-01-04

**Authors:** Isabelle Bekeredjian-Ding, Sandra Foermer, Carsten J. Kirschning, Marijo Parcina, Klaus Heeg

**Affiliations:** 1 Department of Infectious Diseases, Medical Microbiology and Hygiene, University Hospital Heidelberg, Heidelberg, Germany; 2 Institute for Medical Microbiology, University Hospital Essen, Essen, Germany; University of Rochester School of Medicine, United States of America

## Abstract

Poke weed mitogen (PWM), a lectin purified from *Phytolacca americana* is frequently used as a B cell-specific stimulus to trigger proliferation and immunoglobulin secretion. In the present study we investigated the mechanisms underlying the B cell stimulatory capacity of PWM. Strikingly, we observed that highly purified PWM preparations failed to induce B cell proliferation. By contrast, commercially available PWM preparations with B cell activity contained Toll-like receptor (TLR) ligands such as TLR2-active lipoproteins, lipopolysaccharide and DNA of bacterial origin. We show that these microbial substances contribute to the stimulatory activity of PWM. Additional experimental data highlight the capacity of PWM to enable B cell activation by immunostimulatory DNA. Based on these findings we propose that the lectin sensitizes B cells for TLR stimulation as described for B cell receptor ligation and that B cell mitogenicity of PWM preparations results from synergistic activity of the poke weed lectin and microbial TLR ligands present in the PWM preparations.

## Introduction

Due to their immunostimulatory properties plant extracts are commonly used for immune therapy, usually with the aim of inducing an immune response in patients considered to be immunodeficient due to an underlying malignancy. However, despite frequent reports of successful outcomes little is known on the exact mechanisms mediating these beneficial effects.

In the recent past, many groups have described plant-derived substances with immunostimulatory properties. Among them several reports claimed that their effects were based on the activation of Toll-like receptor (TLR)-4, a pattern recognition receptor that mediates proinflammatory immune responses to Gram negative bacteria due to recognition of the lipid A component of the cell wall-associated lipopolysaccharide (LPS). The nature of these substances ranges from proteins such as a 55 kDa protein identified in *Aeginetia indica* [1] to polysaccharides as in *Acanthopanax senticosus*, *Acanthopanax koreanum* and *Carthamus tinctorius L.* [2], [3], [4]. However, many researchers have raised concerns since LPS contamination is easily introduced during the purification procedure, and TLR4 activity may, therefore, not represent an intrinsic property of the plant-derived molecules.

Another plant-derived polysaccharide is poke weed mitogen (PWM), a lectin derived from *Phytolacca americana*. Due to their potent immunostimulatory properties *Phytolacca americana* extracts are used in patients with infections and cancer. Furthermore, PWM is commonly used for B cell assays *in vitro*. Along with “*SAC*”, a suspension of formalin-fixed *S. aureus* cells, it is the most frequently used stimulatory reagent in assays performed for diagnostic purposes, e.g. evaluating B cell function in patients with suspected immunodeficiency. Based on previous studies it is clear that PWM potently stimulates B cell proliferation and immunoglobulin production [5], [6]. Furthermore, combination of *SAC* and PWM results in a synergistic increase in B cell activation and has therefore been proposed as a better control stimulus for B cell assays [5].

Previously, we showed that *SAC* stimulation results from synergistic effects of *S. aureus* surface protein A and bacterial Toll-like receptor (TLR) ligands, such as TLR2-active lipoproteins [7]. By contrast, the effects of PWM have been attributed to the crosslinking of glycoproteins on the B cell surface [8], [9]. More recently, however, one report claimed that commercially available PWM preparations contain contaminant LPS, which contributes to immune cell activation [10]. However, we previously demonstrated that human peripheral blood B cells lack relevant TLR4 expression and are unresponsive to LPS stimulation [11], [12]. Therefore, TLR4 activity cannot readily explain the mitogenic activity of the poke weed lectin on human B cells. In views of its frequent utilization, in the present study we sought to identify its mode of action.

## Materials and Methods

### Mice and murine B cell isolation

**Figure 1:**
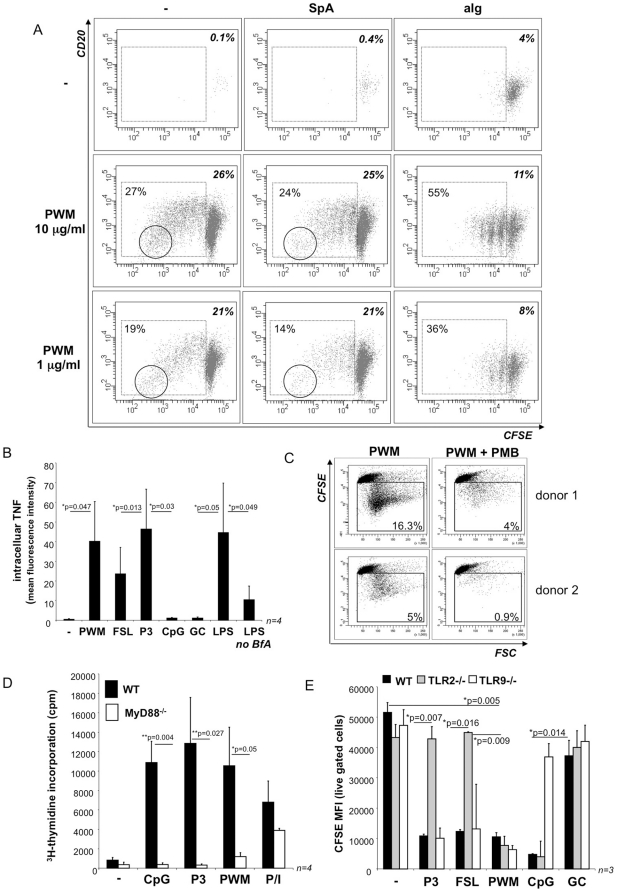
TLR-dependency of Poke weed mitogen (PWM)-induced cell stimulation. **A: B cell proliferation.** Human CD19+ peripheral blood B cells were stained with CFSE and stimulated with 1 or 10 µg/ml PWM in the presence or absence of 5 µg/ml *Staphylococcus aureus* protein A (SpA) or 5 µg/ml anti-human Ig F(ab′)_2_ fragments (aIg) as B cell receptor stimuli, with SpA or aIg alone, or left unstimulated. After 5 days B cells were harvested, stained with anti-CD20 and live gated cells were analyzed for CFSE dilution. The percentages of live gated cells (upper right corner) and proliferating cells (left) are indicated in each diagram. The diagrams depict the results from one representative experiment of n = 3. **B: TNF-induction.** Human PBMC were stimulated for four hours with or without PWM, TLR2 ligands FSL-1R (FSL) and Pam_3_CSK_4_ (P3), phosphorothioate-modified DNA ODN CpG 2006 (CpG) and 2006 GC (GC) and LPS in the presence of Brefeldin A (BfA) or with LPS in the absence of BfA. Subsequently, intracellular staining was performed with an anti-human TNF mAb and TNF expression was analyzed by flow cytometry. The results show a summary of the data obtained in n = 4 experiments. Mean values of anti-TNF mean fluorescence intensities are provided ± SEM. **C: Antagonization with Polymyxin B.** CFSE-stained human CD19+ B cells were stimulated with PWM in the presence and absence of polymyxin B. Proliferation was assessed by flow cytometric analysis of CFSE dilution on day 5. The results obtained in two representative donors of n = 3 are shown. **D: MyD88-dependency of B cell stimulation.** B220+ B cells were isolated from the spleens of MyD88^−/−^ mice and their wild type counterparts. B cells were stimulated with CpG, P3, PWM and PMA/Ionomycin (P/I) and harvested after 72 hours and a 18 hour pulse with ^3^H-thymidine. The diagram shows the average values in counts per minute (cpm) of n = 4 experiments ± SEM. **E: TLR-dependency.** Wild type, TLR2−/− and TLR9−/− B cells were isolated from murine spleen with anti-CD19 microbeads, labelled with CFSE and stimulated with TLR2 ligands Pam_3_CSK_4_ (P3) and FSL-1R (FSL), PWM (10 µg/ml), TLR9 ligand CpG ODN 1668 or 1668 GC control ODN. After 4 days B cell proliferation (CFSE dilution) was quantified by flow cytometry. The diagram depicts the mean fluorescence intensity (MFI) for CFSE in live gated cells as mean value ± SEM from n = 4 experiments. Note that low MFI corresponds to strong proliferation while high MFI values reveal absence of proliferation.

The use of mice was approved by the interfacultary biomedical research facility and the governmental agency at the Regierungspraesidium Karlsruhe (written permit #T4/08). TLR2^−/−^ [13] and MyD88^−/−^ mice [14] were backcrossed to C57Bl/6 background for >6 generations. For B cell isolation spleens were passed through a mesh, filtrated and B cells isolated with anti-B220 (Fig. 1D) or anti-CD19 microbeads (Fig. 1E) (Miltenyi Biotec, Bergisch-Gladbach, Germany). B cell purity was 95±3%. B cells were resuspended in culture medium (RPMI 1640 (Invitrogen, Karlsruhe, Germany) supplemented with 10% FCS (BioWest, Nuaille, France), 1% Penicillin-Streptomycin, 1% HEPES buffer and 1∶50000 β-mercaptoethanol (all from Sigma, Munich, Germany)).

### Human PBMC and B cell isolation

In accordance with the declaration of Helsinki venous blood was drawn from healthy volunteers after verbal and written information on potential adverse events of the blood draw, research goals and measures securing the anonymity of the donors. We obtained written informed consent before venous puncture. The procedure was approved by the ethics commission of the Medical Faculty of the University of Heidelberg, Germany (written approval #157/2006). PBMC were isolated from heparinised venous blood by Ficoll gradient centrifugation. B cells were isolated with anti-CD19 microbeads (Miltenyi). B cell purity was 98±1%. B cells were resuspended in culture medium and incubated over night before stimulation to obtain resting B cells.

### Cell Stimulation

The stimulatory reagents were used at the following concentrations, unless otherwise indicated: all ODN were used at (1 µM) and purchased from MWG Biotech, Munich Germany: CpG ODN 1668 PTO (5′-tccatgttcctgatgct-3′; full-length PTO), CpG ODN 2006 PTO (5′-tcgtcgttttgtcgttttgtcgtt-3′; full-length PTO), GpC ODN 2006 PTO (5′-tgctgcttttgtgcttttgtgctt-3′; full-length PTO), CpG ODN 2006 PO (5′-TCGTCGTTTTGTCGTTTTGTCGTT-3′); Pam_3_CSK_4_ and FSL-1R (HEK293 and monocytes: 200 ng/ml; B cells: 1 µg/ml)) (EMC Microcollections, Tübingen, Germany), MALP-2 (HEK293: 25 ng/ml; B cells: 1 µg/ml) (Alexis Biochemicals, Loerrach, Germany), highly purified LPS from *Salmonella* (gift from U. Seidel, Research Center Borstel, Germany), SpA (Amersham, Uppsala, Sweden), anti-human IgM+IgG+IgA F(ab′)_2_ fragments (Dianova, Hamburg, Germany), PMA (1 µM) and ionomycin (0.5 µg/ml) and polymyxin B (250 U/ml), all from Sigma. The PWM preparations used in this study were purchased from Sigma (Lot. O L8777-5MG (Lot. # not available, purchased in 2005), Lot. A: L8777-5MG #045K7550, Lot. B: L8777-5MG #077K76801, Lot. C: L9379-40MG #038K7680V) and from EY Laboratories, San Mateo, CA, USA (Lot. D: L1901 #260529-5) and used at 1 and 10 µg/ml as indicated. The tomato (*Lycopersicon esculentum*)-derived lectin was purchased from EY laboratories (Lot# 270624-1). As for PWM Lot. D this lot was nearly free of contaminants in LAL, 16 s rDNA PCR and TLR2 assays (data not shown). For neutralisation of human TLR2-transfected HEK293 cells and B cells were pretreated with 10 µg/ml anti-TLR2 mAb for 30 minutes (Genentech, South San Francisco, CA, USA) or the respective isotype control, i.e. murine IgG_1κ_ (Dako, Hamburg, Germany).

For DNAse treatment 20 µl PWM stock 0.1 mg/ml were incubated with or without 1 unit RNAse-free DNAse (Roche, Mannheim, Germany) and 2 µl 10× incubation buffer provided by the manufacturer over night at 37°C. For enzyme inactivation probes were heated to 75°C for 10 minutes and then frozen until further use. N,N-di-acetylchitobiose and N,N,N-tri-acetylchitotriose were purchased from Sigma and used at 400 µg/ml.

### Proliferation assays

For analysis of ^3^H-thymidine incorporation B cells were plated at 0.25×10*6/ml and stimulated for 72 hours, and pulsed 16–18 hours before harvest with 1 µCi/well ^3^H-methyl-thymidine. Proliferation was quantified by detected counts per minute (cpm). For CFSE dilution assays B cells were stained with 10 µM CFSE (Invitrogen), plated at 1×10*6 B cells/ml, stimulated and analyzed by flow cytometry on day 4 or 5.

### Transfection of HEK293 cells and IL-8 ELISA

For IL-8 measurements HEK293 cells were transfected with pTLR2 [15] 200 ng/well complexed with lipofectamine 2000 (Invitrogen) as previously described [7]. Cellular supernatants were harvested 24 hours after stimulation and analyzed for IL-8 by ELISA (BD Opteia, BD Bisociences, Heidelberg, Germany).

### LAL assay

Endotoxin content was determined by kinetic chromogenic limulus amoebocyte lysate (LAL) test (Bio Whittaker, Verviers, Belgium) performed according to the supplier's instructions.

### Immunofluorescence

For immunofluorescence analysis 0.5×10*6/well HEK293 cells were grown over night on poly-lysine D (Sigma) coated coverslips in 24-well plates. Cells were transfected with pTLR2 or with lipofectamine alone and stained for TLR2 24 hours after transfection. For immunofluorescence analysis cells were fixed with PBS/4% PFA, stained with anti-TLR2 mAb or the murine IgG_1κ_ isotype control in Fix & Perm Medium B (Invitrogen), mounted in 0.1 µM DAPI (Invitrogen)-containing PBS/glycerol and stored at 4°C until analysis on a Leica DMI 6000B (Leica Microsystems, Mannheim, Germany).

### Flow cytometry

All experiments were performed on a FACSCanto device (BD Biosciences) and analyzed using the FACS Diva software. Only live gated cells were subjected to analysis. Analysis of intracellular TNF expression in human monocytes was performed as previously described [16]; briefly, PBMC (2×10*6/ml) were incubated with stimulatory reagents with/without Brefeldin A (1 µg/ml, Sigma) for 4 hours, fixed, permeabilized, stained with PE-conjugated anti-human TNF (Becton Dickinson) and analyzed by flow cytometry. Monocytes were gated based on forward scatter (FSC)/side scatter (SSC) properties.

### Amplification of bacterial ribosomal DNA and sequencing

Isolation of DNA was performed with the QIAamp DNA Blood Mini Kit (Qiagen, Hilden, Germany). For amplification of 16S ribosomal DNA PCR was performed with the following degenerate primers: forward 5′-AGA GTT TGA TCM TGG CTC AG-3′; reverse 5′-CCG TCA ATT CMT TTR AGT TT-3′ and the FastStart TaqPolymerase Mix (Roche, Mannheim, Germany). Annealing: 53°C 1 min., Extension: 72°C 1.5 min., 35 cycles. The expected fragment size (*E. coli*) is 919 bp. 16S rDNA-PCR products were purified using the PCR purification kit from Qiagen, Hilden, Germany and sequenced at GATC Biotech AG, Konstanz, Germany. Sequences were blasted against the NCBI database.

## Results

### Poke weed mitogen activity is modulated by B cell receptor ligation

Despite the mechanism of action remains unclear, researchers and clinicians frequently use PWM as a B cell-specific stimulatory reagent with the aim of inducing polyclonal B cell proliferation and immunoglobulin (Ig) secretion. As shown in Figure 1A PWM triggers a long lasting B cell expansion visualized by CFSE dilution and concomitant terminal differentiation into antibody-secreting cells (ASC) (reflected by loss of CD20 expression). It should, however, be noted that high concentrations of PWM ranging from 1–10 µg/ml are required to elicit this response.

Next, we assessed the co-stimulatory potential of the PWM preparation. To this end, we combined PWM with two distinct B cell receptor (BCR) stimuli, i.e. *Staphylocoocus aureus* protein A (SpA) and anti-human IgM+IgG+IgA F(ab′)_2_ fragments (anti-Ig). The results showed that the percentage of PWM-induced proliferating cells and the overall B cell survival were almost unaffected by SpA (Fig. 1A). In marked contrast, BCR crosslinking with anti-Ig resulted in an increased percentage of proliferating B cells and a concomitant decrease in B cell survival (Fig. 1A). Moreover, stimulation with PWM or PWM with SpA not only supported prolonged B cell division cycles but also promoted plasma blast differentiation coinciding with loss of CD20 surface expression in the cells with the highest CFSE dilution (Fig. 1A). In contrast, combination of PWM with anti-Ig did not support terminal differentiation and prevented repeated cell division (≥2 generations) despite the increased cellular turnover (Fig. 1A). BCR crosslinking with anti-Ig, thus, interferes with PWM-triggered effector functions.

### PWM induces TNF in monocytes and PWM-induced B cell expansion is blocked by TLR4 antagonist polymyxin B

The finding that PWM displayed co-stimulatory activity when combined with a BCR stimulus prompted us to ask whether the PWM preparations contained TLR ligands, e.g. microbial substances well-known to synergize with BCR signaling [7], [17], [18]. This assumption was further supported by the finding that PWM activated human monocytes (Fig. 1B). PWM-induced TNF expression was observed in a flow cytometric assay routinely used to detect minute amounts of microbial contaminants in cell culture reagents [16]. We concluded that low concentrations of microbial molecules such as LPS could account for this finding. Furthermore, PWM-induced B cell proliferation could be inhibited in the presence of polymyxin B, a cationic detergent frequently employed to antagonize LPS (Fig. 1C).

### PWM activates B cells in a MyD88-dependent manner

In line with this observation PWM-triggered murine B cell proliferation was found to depend on MyD88, the key adaptor molecule in TLR signaling: in contrast to the wild type B cells MyD88^−/−^ B cells failed to proliferate in response to PWM albeit proliferation could be induced by PMA and ionomycine (Fig. 1D). However, MyD88-deficiency is associated with attenuated B cell responses [19] and MyD88^−/−^ B cells were, thus, also less responsive to stimulation with PMA and ionomycine than wild type B cells. We, therefore, limited our conclusions to the statement that a role for Toll-like receptor (TLR) ligands in PWM-driven B cell proliferation could not be excluded. Experiments using B cells deficient in single TLRs, i.e. purified from TLR2- or TLR9-deficient mice, did not display any relevant deficit in their response to PWM albeit selective unresponsiveness to TLR2- or TLR9 ligands, respectively, was confirmed (Fig. 1E). This finding was compatible with previously described endotoxin (LPS) contamination in PWM preparations [10] or a redundant contribution of multiple TLR ligands to B cell proliferation.

### Proliferation of human peripheral blood B cells is triggered via TLR2 or TLR9 but not by TLR4 ligand lipopolysaccharide

**Figure 2:**
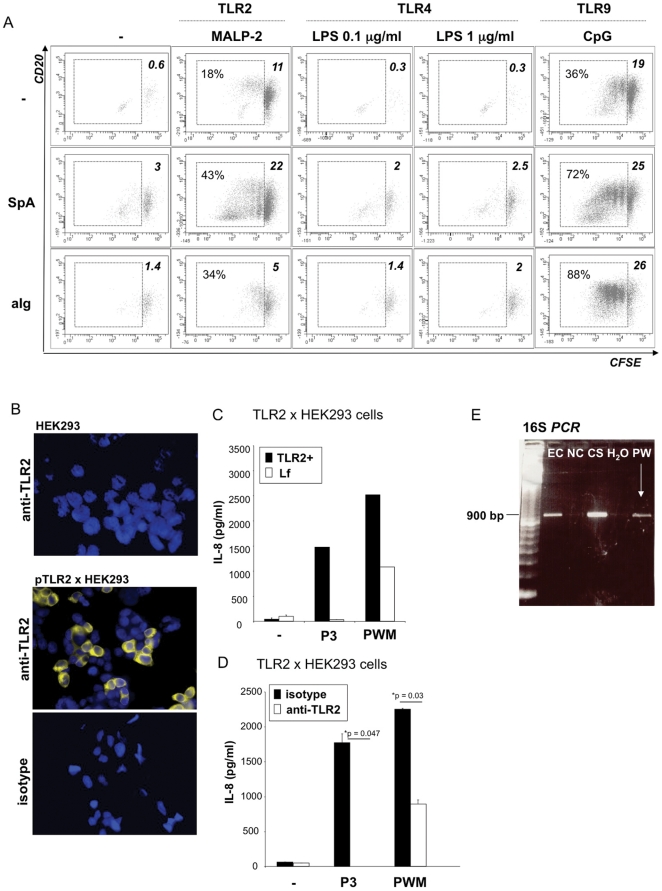
Contribution of TLR ligands to B cell activation. **A: Stimulation of human B cells with TLR ligands.** CFSE-labelled CD19+ human B cells were stimulated with or without TLR ligands (TLR2 ligand MALP-2, TLR4 ligand LPS (0.1 and 1 µg/ml) or TLR9 ligand CpG ODN 2006 (CpG)) and/or BCR stimuli (5 µg/ml SpA or anti-Ig (aIg)). After 5 days cells were harvested, stained with anti-CD20 and analyzed by flow cytometry. The graphs depict cell survival (percentage of live gated cells; upper right angle) and cell proliferation (CFSE dilution; % proliferating cells of live gated cells (left) where indicated). The experiment shown is representative of n≥3 experiments. **B–D: Assessment of TLR2 activity in PWM preparations.** HEK293 cells were transfected with pTLR2 or lipofectamine alone (Lf). **B:** non-transfected and pTLR2-transfected HEK293 cells were stained for TLR2 expression with anti-TLR2 mAb or the respective isotype control as indicated. **C:** HEK293 cells transfected with pTLR2 or Lf only were stimulated with Pam_3_CSK_4_ (P3) or PWM (10 µg/ml). After 24 hours cellular supernatants were collected and analyzed for IL-8 secretion. One representative experiment of n≥3 experiments is shown. **D:** HEK293 cells transfected with pTLR2 were pretreated with anti-TLR2 mAb or the isotype control before stimulation with Pam_3_CSK_4_ (P3) or PWM (10 µg/ml). IL-8 was quantified in the 24 hour supernatants. **E: 16S rDNA PCR.** DNA isolation and PCR amplification of bacterial 16S ribosomal DNA from DNA from *E. coli* (EC), a negative clinical specimen (NC), a positive clinical sample (CS) and the PWM preparation (PW) or the water control (H_2_O), with an expected PCR fragment size of approximately 900 bp.

To assess a possible contribution of TLR-stimulatory substances in PWM-induced human B cell activation, we stimulated human B cells with ligands for TLR2 (MALP-2), TLR4 (LPS) and TLR9 (CpG ODN) in the presence or absence of BCR crosslinking with SpA or anti-Ig. After 5 days B cell survival and proliferation were detectable after exposure of B cells to TLR2 and TLR9 ligands and were more pronounced when the TLR agonists were combined with SpA and/or anti-Ig (Fig. 2A). In contrast, survival and proliferation above the levels observed in unstimulated B cells were not observed when B cells were treated with LPS at varying concentrations (Fig. 2A). This indicated that LPS contamination cannot account for human B cell activity of PWM preparations, although it could contribute to murine B cell activation as suggested in [10].

### PWM preparations contain TLR2 ligands

This prompted us to ask whether PWM contained other TLR ligands. To test for TLR2 activity we transfected HEK293 cells with or without a human TLR2 expression plasmid (Fig. 2B), and challenged these cells with PWM or a synthetic triacylated lipopeptide, namely Pam_3_CSK_4_. TLR2-dependency of lipopeptide activity indicated specificity of the assay (Fig. 2C). Interestingly, this lot of PWM (Lot. 0) induced basal IL-8 secretion from non-transfected HEK293 cells. This indicated that the PWM preparation (Lot. 0) contained immune stimulatory components acting independently of TLR2, a result well compatible with conserved PWM responsiveness in TLR2-deficient murine B cells (Fig. 1E). Nevertheless, PWM-induced IL-8 production was almost doubled in the presence of TLR2 expression (Fig. 2C) and, when compared to the isotype control, reduced by approximately 50% by an anti-TLR2 neutralizing monoclonal antibody (mAb) (Fig. 2D). This proved the presence of TLR2-active substances within the PWM preparation.

### PWM contains bacterial DNA

Since TLR9-dependent sensing of bacterial DNA is a potent inductor of B cell proliferation (Fig. 2A) we also analyzed the bacterial DNA content of PWM preparations. To this end, we amplified ribosomal bacterial 16S DNA by PCR. Our results showed that bacterial DNA was detectable in PWM preparation (Lot. 0) (Fig. 2E). Sequence analysis of the 16S rDNA sequence revealed that the sequence corresponds to *Propionibacterium acnes* with 99% homology. Bacterial DNA might, thus, represent an additional stimulatory PWM component.

### Neutralization of TLR2 diminishes the proliferative response to PWM in human B cells

**Figure 3:**
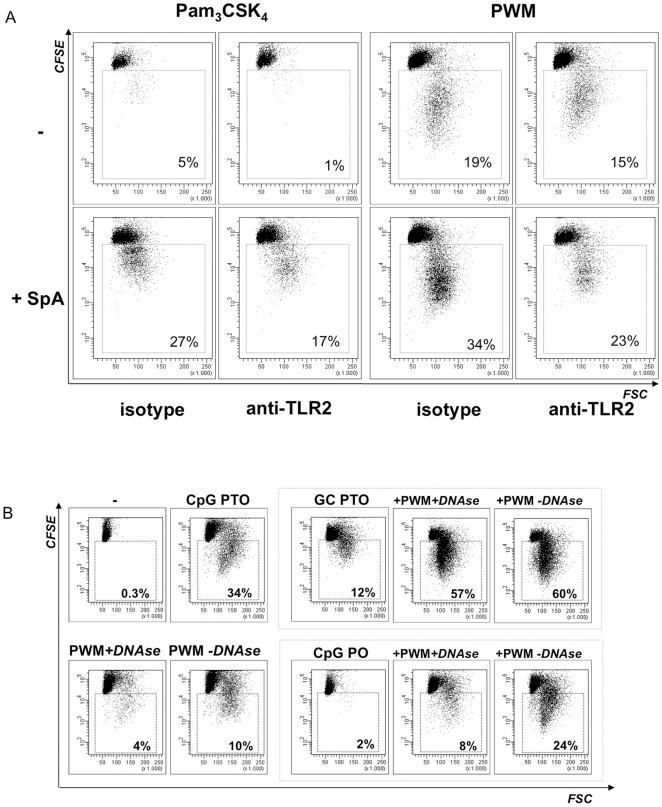
Synergistic effects of TLR ligands with poke weed lectin. **A: Effect of TLR2 blockage on human B cell proliferation in response to PWM.** CD19+ human peripheral blood B cells were labelled with CFSE, preincubated with anti-TLR2 mAb or the corresponding isotype control (murine IgG_1κ_), and stimulated with Pam_3_CSK_4_ or PWM (10 µg/ml) ± SpA (5 µg/ml) for 5 days. Proliferation was assessed by CFSE dilution. The gate denotes the percentage of live gated proliferating B cells as depicted. One representative experiment of n = 3 is shown. **B: Synergistic effects of PWM and immunostimulatory DNA.** Human B cells were stained with CFSE for assessment of B cell proliferation. Stimulation was performed with CpG ODN 2006 (PTO), DNAse- or mock (reaction buffer only)-treated PWM, 2006 GC (PTO) or CpG 2006 (PO) and combinations thereof as indicated. The graphs show the results from one representative experiment from n≥3. The percentage of proliferating B cells is indicated in the graphs.

To assess the relevancy of the TLR2 activity present in the PWM preparations we stimulated human B cells with Pam_3_CSK_4_ or PWM in the absence or presence of the TLR2-blocking mAb or the isotype control already used in Fig. 3A. To better visualize TLR2-mediated B cell activity both stimuli were combined with SpA, that we previously identified as an important enhancer of TLR2 recognition in B cells [7]. In contrast to the experiments performed with murine B cells the results revealed an important contribution of TLR2 activity to PWM-induced proliferation of human B cells: the percentage of proliferating B cells was reduced under TLR2-neutralizing conditions in both Pam_3_CSK_4_- and PWM-stimulated B cells in the presence and absence of SpA (Fig. 3A). Nevertheless, these data also indicated that other TLR2-independent stimuli are involved in PWM-induced B cell activation.

### DNAse treatment of PWM reduces the B cell stimulatory capacity of PWM

To assess the impact of TLR9 ligands on B cell mitogenicity of PWM preparations we treated PWM Lot. 0 with DNAse and tested these fractions in regards to mitogenic activity on human B cells. Proliferative activity was markedly reduced when compared to B cells stimulated with PWM incubated with reaction buffer but no enzyme (Fig. 3B). By contrast, DNAse treatment of TLR2-active lipopeptide Pam_3_CSK_4_ did not alter its stimulatory capacity, nor did addition of heat-inactivated DNAse to the medium affect B cell responsiveness to stimulation (data not shown). We concluded that bacterial DNA contributes to PWM-triggered stimulation of human B cells.

### PWM enables B cell stimulation with atypical DNA ODN

According to a common view DNA ODN lacking the phosphorothioate (PTO) modification and naked bacterial DNA display reduced B cell activity (Fig. 3B) and [20]. Thus, our findings raised the question how the stimulatory effect of the PWM-contained bacterial DNA was conferred. We proposed that the lectin might facilitate bacterial DNA-driven B cell activation. To test this hypothesis we combined PWM with DNA oligodeoxynucleotides (ODN) that by themselves display comparably low mitogenic activity (Fig. 3B), e.g. a PTO-modified GpC control ODN that lacks the TLR9-stimulatory CpG motif (GpC PTO) and a CpG ODN lacking the PTO modification previously shown to be essential for cellular uptake and activity of CpG ODN [21] (CpG PO). CFSE dilution experiments revealed that these “less active” ODN displayed B cell activity when combined with PWM and synergistically enhanced the mitogenic property of the PWM, independent of prior DNAse treatment. As shown in Fig. 3B the comparably weak proliferative response to PTO-modified GpC ODN was strongly augmented in the presence of PWM; and unmodified phosphodiester CpG ODN gained stimulatory activity and augmented PWM-induced proliferation. Altogether, these data demonstrated that the PWM preparation contains substances that promote the activity of otherwise inactive synthetic DNA ODN and/or facilitate TLR9-mediated B cell activation.

### Mitogenic activity correlates with presence of TLR ligands in PWM preparations

**Figure 4:**
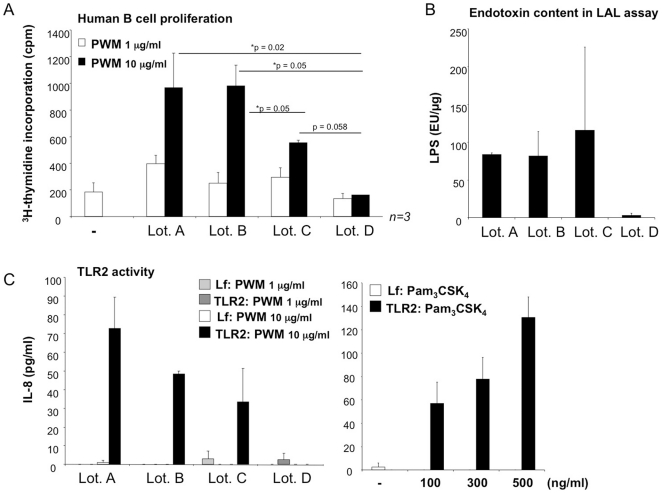
Detection of TLR ligands in PWM preparations. **A:** Human B cells were stimulated with different lots of PWM. Proliferation rates were quantified by 3H-thymidine incorporation (cpm = counts per minute). The diagram shows the mean values ± SEM from n = 3 experiments. **B:** LAL-assay was performed to quantify the LPS content in PWM Lot. A–D. The diagram depicts the mean values ± standard deviation obtained by testing in quadruplicates. **C:** TLR2 activity was assessed by measuring IL-8 concentrations in the supernatants of pTLR2-transfected or non-transfected HEK293 cells stimulated with different lots of PWM (left) or Pam_3_CSK_4_ (right) at the concentrations indicated. One representative experiment performed in triplicates of n = 2 is shown.

To further assess the impact of TLR-active substances in PWM preparations on PWM-induced B cell activation we compared four different commercially available PWM preparations in regards to their mitogenic potential and TLR stimulation. The results showed that PWM Lot. A, B and C were superior to Lot. D in the induction of human B cell proliferation (Fig. 4A). This increase in stimulatory activity correlated with higher endotoxin content determined by limbulus assay (LAL assay) (Fig. 4B) and with stronger TLR2-activity in the HEK293 system (Fig. 4C). Notably, TLR2 activity lay below the detection threshold when measured in 1 µg/ml PWM, but was detectable at a concentration of 10 µg/ml. Comparable levels of IL-8 secretion were achieved by stimulation of transfected HEK293 cells with 300 ng/ml of TLR2 ligand Pam_3_CSK_4_. Interestingly, when compared to Lot. 0 in these more recently purchased lots of PWM the levels of TLR2 activity were overall lower, IL-8 induction was restricted to TLR2-transfected HEK293 cells and we did not amplify bacterial DNA by PCR. In fact, in Lot. D, which was purchased as “highly purified” PWM, even LPS content and TLR2 activity were negligible. This PWM preparation also failed to induce B cell proliferation (Fig. 4A), thus, corroborating the concept that PWM-induced proliferation is driven by TLR ligands.

### (GlcNAc)_3_-binding lectins costimulate B cell proliferation induced by immune stimulatory DNA

**Figure 5:**
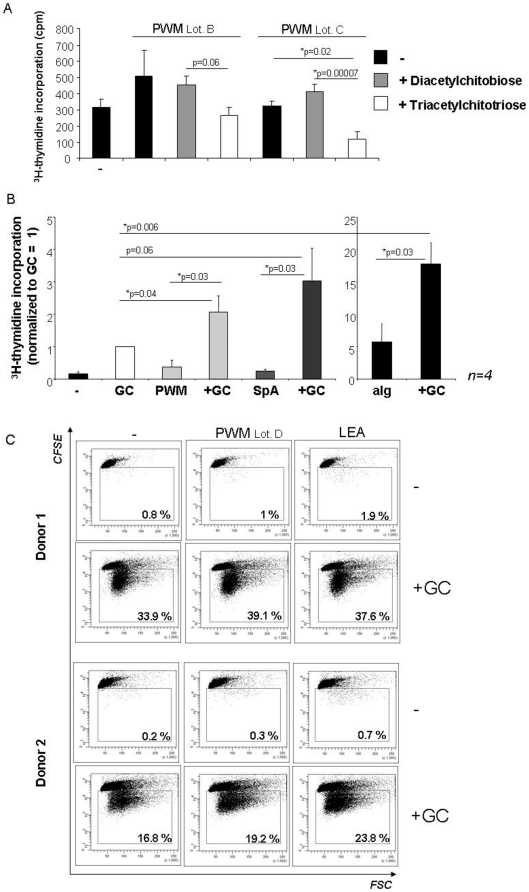
Contribution of the lectin component. **A:** Human B cell proliferation in response to PWM (Lot. B and C) in the presence or absence of N,N-di-acetylchitobiose or N,N,N-tri-acetylchitotriose was assessed by ^3^H-thymidine incorporation given in counts per minute (cpm). The mean values ± SEM from one representative experiment performed in triplicates of n = 2 is shown. **B:** Human CD19+ B cells were left unstimulated or stimulated with 0.25 µM GpC PTO ODN (GC) and/or 10 µg/ml of PWM, 5 µg/ml SpA or 10 µg/ml anti-Ig (aIg) for 72 hours. Proliferation was quantified by ^3^H-thymidine incorporation. The diagram shows the means from n = 4 experiments ± SEM. The values obtained were normalized to GC = 1 (710±280 cpm = mean ± SEM). **C:** Human CD19+ B cells were stained with CFSE and stimulated with highly purified PWM (Lot. D) or *Lycopersicon esculentum* lectin (LEA) with or without GpC PTO ODN (GC). Proliferation was quantified by CFSE dilution on day 4. The percentage of proliferating cells is provided in each dot plot. The results from two independent donors of n = 4 are shown.

Earlier studies have described the (GlcNAc)_3_ carbohydrate specificity of *Phytolacca americana* (PWM) lectin [8]. It was further shown that the (GlcNAc)_3_ binding can be antagonized by N,N-di-acetylchitobiose and N,N,N-tri-acetylchitotriose. We, therefore, used these substances to assess the contribution of the lectin component within the PWM preparation. As shown in Figure 5A the effect of N,N-di-acetylchitobiose was negligible, but N,N,N-tri-acetylchitotriose inhibited PWM-triggered B cell proliferation, thus confirming the contribution of (GlcNAc)_3_ binding to mitogenicity.

Based on these findings we hypothesized that the lectin component in PWM might act as a sensitizer for B cells towards microbial ligands as previously described for BCR ligation [7], [22]. To test this hypothesis human B cells were stimulated with highly purified PWM (Lot. D) or a BCR stimulus (SpA or anti-Ig) in the presence and absence of GpC PTO, as introduced in Fig. 3B. The results indicated that, similarly to SpA and anti-Ig, PWM increases the proliferative activity of immune stimulatory DNA (Fig. 5B).

Lastly, a non-related lectin with (GlcNAc)_3_ specificity from *Lycopersicon esculentum* (tomato) [23] was used to corroborate our findings. Albeit by themselves highly purified preparations of PWM and tomato lectins displayed very little activity, comparative analysis in CFSE dilution assays demonstrated that both lectins augmented B cell proliferation induced by GpC PTO (Fig. 5C). Lectin-typical carbohydrate binding may, thus, co-stimulate B cell activation by nucleic acid-sensing pattern recognition receptors.

## Discussion

The results obtained in this study indicate that the mitogenic activity of PWM on human B cells is based on the synergistic action of the poke weed lectin and the co-purified bacterial TLR ligands specific for TLR2 and TLR9. DNA contained in the PWM preparation was found to be of bacterial origin, since ribosomal bacterial DNA was amplified with specific PCR primers when PWM-extracted DNA was used as a template (Fig. 2E). Similarly, TLR2-specific ligands (Fig. 2C and 3A) are most likely derived from bacterial lipoproteins, considering that, to date, there is no evidence for plant-derived TLR2 ligands. Although we did not follow this up, it is very likely that the PWM preparations contain low concentrations of other bacterial molecules such as peptidoglycan or lipoteichoic acids.

It is well-known that specific bacterial species preferentially interact with plants. *Rhizobium spp.* for example are plant symbionts while others including most *Agrobacterium spp.* and *Burkholderia spp.* are phytopathogens [24]. These genus have a Gram negative cell wall and, thus, carry LPS among other specific microbial molecules that confer bacterial recognition by mammalian pattern recognition receptors. However, despite the attractiveness of the hypothesis, our findings provided no evidence for plant-associated pathogens as a source for TLR ligands and we could not exclude contamination of PWM during the purification procedure: sequencing of the 16S rDNA PCR product in Lot. 0 revealed the presence of DNA from *Propionibacterium acnes*, a typical inhabitant of human skin containing CG-rich TLR9-stimulatory genomic DNA [25].

Taking into consideration that different classes of bacterial molecules are detectable in the PWM preparation, we propose that the endotoxin content in PWM preparations (Fig. 4B and [10]) could originate from inaccuracies in the chemical purification procedure as well as from plant-derived bacteria. Nevertheless, regardless of its origin LPS will contribute to the stimulation of TLR4-carrying immune cells such as human monocytes (Fig. 1B) and murine B cells (Fig. 1E).

However - in contrast to monocytes and murine B cells - human B cells lack expression of the lipopolysaccharide sensors CD14 and TLR4 [12], [18]. Correlating with receptor expression human B cells fail to respond to LPS despite the presence or absence of a BCR stimulus (Fig. 2A). As a consequence, LPS is not an important PWM component conferring mitogenic activity on human B cells although it may act on other leukocyte subsets. Since polymyxin B is a cationic detergent antagonization of PWM-induced B cell activation does not necessarily need to be limited to LPS, but could also involve bacterial lipoproteins or DNA. Nevertheless, PWM-derived LPS may very well contribute to murine B cell stimulation as was recently reported for a polysaccharide isolated from *Acanthopanax senticosus* that activated murine B cells and macrophages in a TLR4-dependent manner [4] and a polysaccharide purified from *Acanthopanax koreanum* that displayed TLR2- and TLR4-dependent B cell stimulatory activity [3].

In contrast to TLR4 stimulation, human peripheral blood B cells respond to TLR2 and TLR9 specific challenge and the response is increased in the presence of BCR stimulation (with SpA or anti-Ig) that was previously found to enable TLR2 activity and to augment the TLR9 response in human B cells (Fig. 2A and [7], [17], [22]). In contrast to TLR2 ligands PWM displays mitogenic activity in the absence of BCR stimulation (Fig. 1A). This indicates that PWM contains stimulatory agents that either circumvent the requirement for BCR ligation or directly engage the BCR. This effect could be attributed to the lectin compound that binds to cell surface N-acetylglucosamine-containing glycoproteins [8], [26]. Lectin-mediated engagement of rather ill-defined surface receptors may, thus, enable TLR2 activity and/or enhance the activity of other TLRs such as the DNA sensor TLR9 [7], [17], [22].

Surprisingly, the combination of PWM with SpA failed to increase proliferation although it was recently suggested that combined stimulation of PWM and *SAC*, a suspension of *S. aureus* cells with high SpA content, provides a stronger and more reliable stimulus in human PBMC [5]. However, the fact that our experiments were carried out using purified human B cells, e.g. in the absence of antigen presenting cells, may account for this difference. Notably, in our experimental setting a slight decrease in the percentage of proliferating B cells observed when SpA was used together with PWM (Fig. 1A) could indicate that SpA and PWM compete for the same B cell binding sites. Again, we can only speculate whether this could involve a subgroup of B cell receptors such as the V_H_3+ BCR targeted by SpA [27]. However, this observation was supported by the finding that BCR stimulation with anti-Ig markedly reduced the duration of proliferation and terminal differentiation in B cells stimulated with PWM (Fig. 1A). This provides the notion that BCR stimulation might interfere with the cellular binding of *Phytolacca americana* lectin.

Taking into consideration that only traces of bacterial DNA and lipoproteins are present in the PWM preparations it is evident that high concentrations of PWM (1–10 µg/ml) are required for B cell stimulation. Moreover, our data suggest that the poke weed lectin enables B cell stimulation by low concentrations of bacterial DNA that by themselves would be ineffective. They further highlight that non-CpG DNA sequences and non-PTO modified ODN display immune stimulatory activity in the presence of PWM (Fig. 3B and 5). Interestingly, similar observations were made when B cell stimulation with non-CpG ODN occurred together with a BCR antigen or when ODN lacking B cell activity were targeted to the BCR [28], [29]: B cell ODN sequence requirements were found to be less stringent in the presence of BCR activation, and uptake of ODN improved.

In the present context, we propose that similarly to a BCR signal PWM sensitizes B cells for pathogen-associated molecular patterns (PAMPs). It can only be speculated whether the positive charge of the *Phytolacca americana*-derived lectin might enable complexation with negatively charged bacterial molecules such as DNA [30]. Similarly to PTO-modification of synthetic ODN or formation of autoimmune complexes as in [28] these complexes could protect DNA from degradation and promote cellular uptake of bacterial DNA. Alternatively, lectin-mediated binding to carbohydrates on cell surface receptors facilitates uptake of these substances and/or cellular activation. Endocytotic uptake, in turn, could provide access to TLR9 and induces intracellular redistribution of TLR9 to the autophagosomes [31], [32].
